# Poly[(μ-1,3-thio­cyanato-κ*N*,*S*)(iso­nicotin­ato-κ*N*,*O*)(ethanol-κ*O*)cadmium(II)]

**DOI:** 10.1107/S1600536812040913

**Published:** 2012-10-06

**Authors:** Tristan Neumann, Julia Werner, Inke Jess, Christian Näther

**Affiliations:** a Institut für Anorganische Chemie, Christian-Albrechts-Universität Kiel, Max-Eyth-Strasse 2, 24118 Kiel, Germany

## Abstract

In the crystal structure of the title compound, [Cd(NCS)(C_6_H_4_NO_2_)(C_2_H_5_OH)]_*n*_, the Cd^2+^ cation is coordinated by one N and two O atoms of two symmetry-related isonicotinate anions, one ethanol mol­ecule and two μ-1,3-bridging thio­cyanate anions in a distorted octa­hedral N_2_O_3_S geometry. The metal cations are μ-1,3-bridged *via* thio­cyanate anions into chains that are further connected into layers parallel to the *ab* plane by bridging isonicotinate anions. The layers are stacked along the *c* axis. The crystal structure is stabilized by O—H⋯O hydrogen bonds.

## Related literature

For general background information, including details of thermal decomposition reactions and magnetic properties, see: Näther & Greve (2003 ▶); Boeckmann & Näther (2010 ▶, 2011 ▶); Wöhlert *et al.* (2011 ▶). For related structures, see: Yang *et al.* (2001 ▶). For a description of the Cambridge Structural Database, see: Allen (2002 ▶).
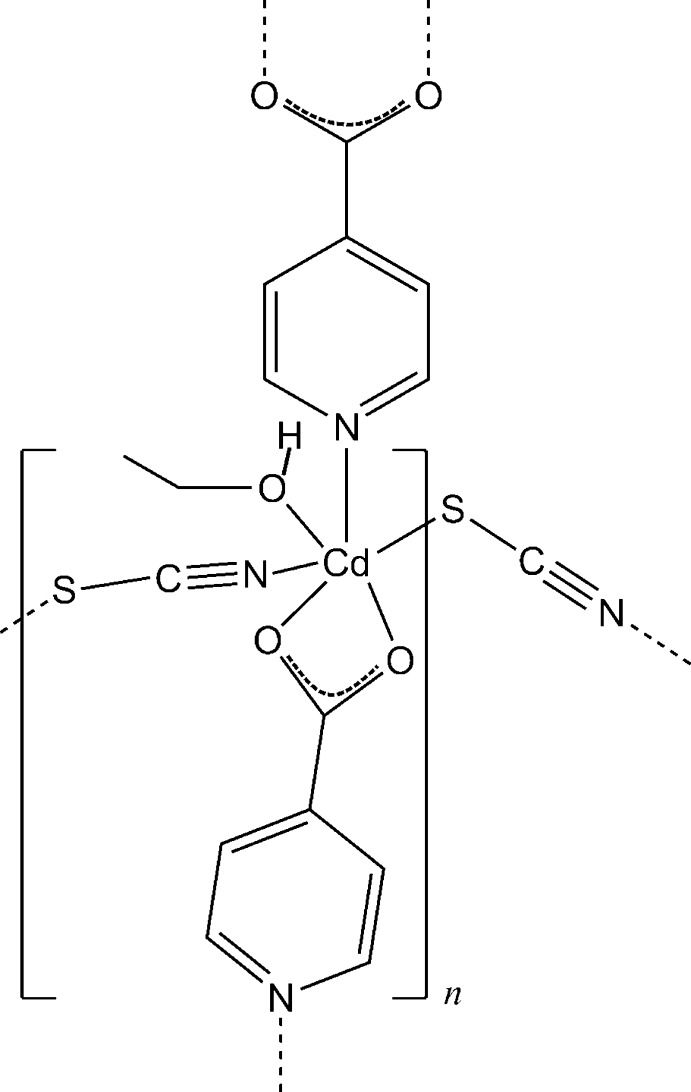


## Experimental

### 

#### Crystal data

[Cd(NCS)(C_6_H_4_NO_2_)(C_2_H_6_O)]
*M* *_r_* = 338.65
Monoclinic, 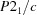

*a* = 5.7778 (2) Å
*b* = 16.1804 (6) Å
*c* = 13.0855 (6) Å
β = 94.685 (3)°
*V* = 1219.24 (8) Å^3^
*Z* = 4
Mo *K*α radiation
μ = 1.96 mm^−1^
*T* = 293 K
0.28 × 0.10 × 0.04 mm

#### Data collection

Stoe IPDS-1 diffractometer
Absorption correction: numerical (*X-SHAPE* and *X-RED32*; Stoe, 2008 ▶) *T*_min_ = 0.803, *T*_max_ = 0.931
17537 measured reflections
2920 independent reflections
2545 reflections with *I* > 2σ(*I*)
*R*_int_ = 0.032

#### Refinement

*R*[*F* ^2^ > 2σ(*F* ^2^)] = 0.026
*wR*(*F* ^2^) = 0.061
*S* = 1.06
2920 reflections
145 parameters
H-atom parameters constrained
Δρ_max_ = 0.38 e Å^−3^
Δρ_min_ = −0.44 e Å^−3^

### 

Data collection: *X-AREA* (Stoe, 2008 ▶); cell refinement: *X-AREA*; data reduction: *X-AREA*; program(s) used to solve structure: *SHELXS97* (Sheldrick, 2008 ▶); program(s) used to refine structure: *SHELXL97* (Sheldrick, 2008 ▶); molecular graphics: *XP* in *SHELXTL* (Sheldrick, 2008 ▶) and *DIAMOND* (Brandenburg, 2011 ▶); software used to prepare material for publication: *publCIF* (Westrip, 2010 ▶).

## Supplementary Material

Crystal structure: contains datablock(s) I, global. DOI: 10.1107/S1600536812040913/bt6838sup1.cif

Structure factors: contains datablock(s) I. DOI: 10.1107/S1600536812040913/bt6838Isup2.hkl

Additional supplementary materials:  crystallographic information; 3D view; checkCIF report

## Figures and Tables

**Table 1:** Hydrogen-bond geometry (Å, °)

*D*—H⋯*A*	*D*—H	H⋯*A*	*D*⋯*A*	*D*—H⋯*A*
O21—H1*O*1⋯O12^i^	0.82	1.89	2.703 (3)	172

Symmetry code: (i) 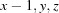.

**Fig. 1.**
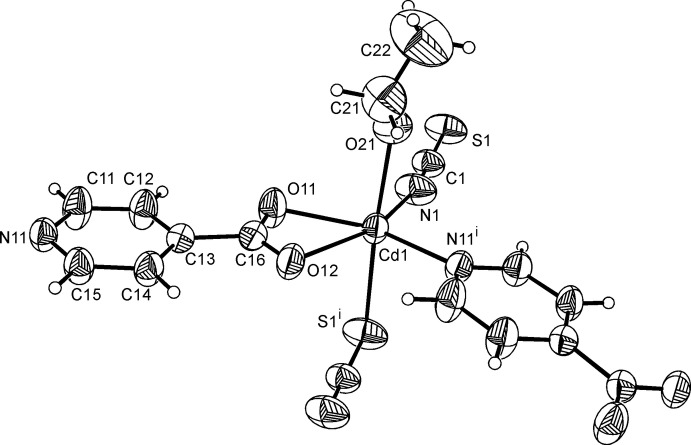
Crystal structure of the title compound with labelling and displacement ellipsoids drawn at the 50% probability level. Symmetry code: i = -x + 1, y + 1/2, -z + 3/2.

**Fig. 2.**
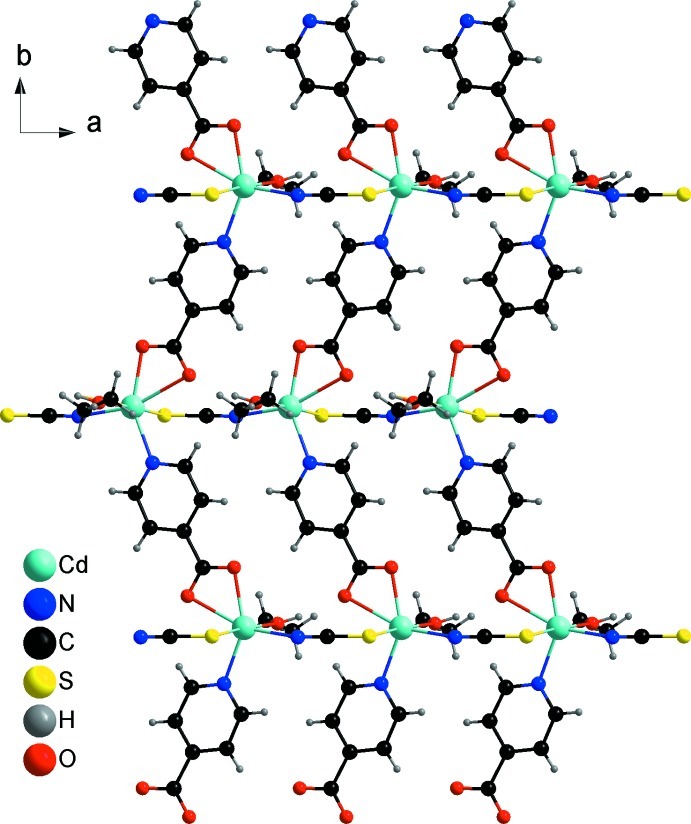
Crystal structure of the title compound with view in the direction of the crystallographic c axis.

## supplementary crystallographic information

### Comment

The structure of the title compound was prepared within a project on the synthesis of transition metal thiocyanato coordination polymers in which the metal cations are µ-1,3 bridged by the anionic ligands (Näther & Greve, 2003; Boeckmann & Näther, 2010, 2011; Wöhlert *et al.*, 2011). In the course of our investigations crystals of the title compound were obtained and characterized by single-crystal X-ray diffraction.

In the crystal structure the cadmium(II) cations are coordinated by one N and two O atoms of two µ-1,3,6 bridging isonicotinato anions which are related by symmetry, one N and one S atom of two symmetry-related µ-1,3 bridging thiocyanato anions and one O atom of an ethanol molecule (Fig. 1). The coordination polyhedron of the cadmium cations can be described as a slightly distorted octahedron (Table 1).

The Cd^2+^ cations are µ-1,3 bridged by thiocyanato anions into chains, which elongate in the direction of the crystallographic *a* axis. These chains are bridged by µ-1,3,6 bridging isonicotinato anions into layers in the direction of the crystallographic *b* axis and the layers are stacked along the crystallographic *c* axis (Fig. 2).

The shortest Cd···Cd distances within the layers amounts to 5.7778 (3) Å and to 9.2393 (4) Å. It must be noted that according to research in the CCDC database (ConQuest Ver.1.14; Allen, 2002) one coordination compound based on Cd(NCS)_2_, isonicotinato anions and thiocyanato anions is known, in which ethanol is exchanged by water. The overall coordination topology is similar but this compound is not isotypic to the title compound (Yang *et al.*, 2001).

The crystal structure is stabilized by an O—H···O hydrogen bond.

### Experimental

Potassium thiocyanate and isonicotinic acid were purchased from Alfa Aesar, Cd(SO_4_)_2_.4H_2_O was obtained from Merck. The Cd(NCS)_2_ was prepared by stirring Ba(NCS)_2_.3H_2_O (3.076 g, 10 mmol) and CdSO_4_.8/3H_2_O (2.566 g, 10 mmol) in water (100 ml). The white precipitate of BaSO_4_ was filtered off and the water was removed from the filtrate by heating. The final product was dried at 80°C. The homogeneity of the product was investigated by X-ray powder diffraction. The title compound was prepared by the reaction of 34.3 mg Cd(NCS)_2_(0.15 mmol) and 36.9 mg isonicotinic acid (0.30 mmol) in 2 ml ethanol at 80°C in a closed 10 ml glass culture tube. After several days colourless needles of the title compound were obtained.

### Refinement

The C—H H atoms were positioned with idealized geometry (methyl H atoms allowed to rotate but not to tip) and were refined isotropically with *U*_iso_(H) = 1.2*U*_eq_(C) for aromatic H atoms (1.5 for methyl H atoms) using a riding model with C—H = 0.93 Å (aromatic H atoms) and with C—H = 0.96 Å (methyl H atoms). The O—H H atom was located in difference map, its bond length set to ideal value of 0.82 Å and finally it was refined using a riding model with *U*_iso_(H) = 1.2*U*_eq_(O).

### Crystal data

**Table:**

[Cd(NCS)(C_6_H_4_NO_2_)(C_2_H_6_O)]	*F*(000) = 664
*M**_r_* = 338.65	*D*_x_ = 1.845 Mg m^−^^3^
Monoclinic, *P*2_1_/*c*	Mo *K*α radiation, λ = 0.71073 Å
*a* = 5.7778 (2) Å	Cell parameters from 17537 reflections
*b* = 16.1804 (6) Å	θ = 2.0–28.0°
*c* = 13.0855 (6) Å	µ = 1.96 mm^−^^1^
β = 94.685 (3)°	*T* = 293 K
*V* = 1219.24 (8) Å^3^	Needle, colourless
*Z* = 4	0.28 × 0.10 × 0.04 mm

### Data collection

**Table:**

Stoe IPDS-1 diffractometer	2920 independent reflections
Radiation source: fine-focus sealed tube	2545 reflections with *I* > 2σ(*I*)
Graphite monochromator	*R*_int_ = 0.032
φ scans	θ_max_ = 28.0°, θ_min_ = 2.0°
Absorption correction: numerical (*X-SHAPE* and *X-RED32*; Stoe, 2008)	*h* = −7→7
*T*_min_ = 0.803, *T*_max_ = 0.931	*k* = −21→21
17537 measured reflections	*l* = −17→17

### Refinement

**Table:**

Refinement on *F*^2^	Primary atom site location: structure-invariant direct methods
Least-squares matrix: full	Secondary atom site location: difference Fourier map
*R*[*F*^2^ > 2σ(*F*^2^)] = 0.026	Hydrogen site location: inferred from neighbouring sites
*wR*(*F*^2^) = 0.061	H-atom parameters constrained
*S* = 1.06	*w* = 1/[σ^2^(*F*_o_^2^) + (0.0288*P*)^2^ + 0.6822*P*] where *P* = (*F*_o_^2^ + 2*F*_c_^2^)/3
2920 reflections	(Δ/σ)_max_ = 0.001
145 parameters	Δρ_max_ = 0.38 e Å^−^^3^
0 restraints	Δρ_min_ = −0.44 e Å^−^^3^

### Special details

**Table:**

Geometry. All esds (except the esd in the dihedral angle between two l.s. planes) are estimated using the full covariance matrix. The cell esds are taken into account individually in the estimation of esds in distances, angles and torsion angles; correlations between esds in cell parameters are only used when they are defined by crystal symmetry. An approximate (isotropic) treatment of cell esds is used for estimating esds involving l.s. planes.
Refinement. Refinement of F^2^ against ALL reflections. The weighted R-factor wR and goodness of fit S are based on F^2^, conventional R-factors R are based on F, with F set to zero for negative F^2^. The threshold expression of F^2^ > 2sigma(F^2^) is used only for calculating R-factors(gt) etc. and is not relevant to the choice of reflections for refinement. R-factors based on F^2^ are statistically about twice as large as those based on F, and R- factors based on ALL data will be even larger.

### Fractional atomic coordinates and isotropic or equivalent isotropic displacement parameters (Å^2^)

**Table:**

	*x*	*y*	*z*	*U*_iso_*/*U*_eq_	
Cd1	0.14587 (3)	0.856111 (10)	0.668028 (15)	0.04089 (7)	
N1	−0.1966 (4)	0.8771 (2)	0.5822 (2)	0.0667 (8)	
C1	−0.3839 (5)	0.87582 (19)	0.5463 (2)	0.0492 (6)	
S1	−0.65079 (13)	0.87510 (7)	0.49397 (6)	0.0662 (2)	
N11	0.7272 (4)	0.48256 (13)	0.77586 (18)	0.0432 (5)	
C11	0.5200 (6)	0.49095 (17)	0.7245 (3)	0.0578 (8)	
H11	0.4417	0.4437	0.7005	0.069*	
C12	0.4168 (5)	0.56691 (17)	0.7052 (2)	0.0540 (7)	
H12	0.2722	0.5702	0.6687	0.065*	
C13	0.5275 (4)	0.63720 (15)	0.7401 (2)	0.0396 (5)	
C14	0.7445 (5)	0.62913 (15)	0.7913 (2)	0.0480 (6)	
H14	0.8275	0.6757	0.8145	0.058*	
C15	0.8372 (5)	0.55133 (16)	0.8077 (2)	0.0490 (6)	
H15	0.9834	0.5466	0.8426	0.059*	
C16	0.4083 (5)	0.71974 (15)	0.7252 (2)	0.0426 (5)	
O11	0.2091 (4)	0.71926 (12)	0.67857 (18)	0.0592 (5)	
O12	0.5052 (3)	0.78408 (11)	0.75867 (17)	0.0510 (5)	
O21	−0.0682 (4)	0.84635 (14)	0.81279 (16)	0.0572 (5)	
H1O1	−0.2025	0.8310	0.7996	0.086*	
C21	0.0069 (7)	0.8386 (3)	0.9186 (3)	0.0778 (11)	
H21A	0.1454	0.8720	0.9327	0.093*	
H21B	0.0497	0.7815	0.9324	0.093*	
C22	−0.1619 (11)	0.8630 (4)	0.9880 (4)	0.129 (2)	
H22A	−0.0982	0.8552	1.0574	0.193*	
H22B	−0.2990	0.8298	0.9757	0.193*	
H22C	−0.2010	0.9201	0.9771	0.193*	

### Atomic displacement parameters (Å^2^)

**Table:**

	*U*^11^	*U*^22^	*U*^33^	*U*^12^	*U*^13^	*U*^23^
Cd1	0.03291 (10)	0.03170 (10)	0.05680 (12)	−0.00051 (7)	−0.00408 (7)	0.00070 (8)
N1	0.0336 (12)	0.098 (2)	0.0675 (16)	0.0051 (13)	−0.0028 (11)	0.0164 (15)
C1	0.0382 (14)	0.0617 (17)	0.0476 (14)	0.0037 (12)	0.0028 (11)	0.0077 (12)
S1	0.0357 (3)	0.1101 (7)	0.0514 (4)	−0.0010 (4)	−0.0046 (3)	0.0062 (4)
N11	0.0401 (11)	0.0317 (10)	0.0569 (13)	0.0011 (8)	−0.0020 (10)	−0.0014 (9)
C11	0.0541 (17)	0.0349 (13)	0.080 (2)	−0.0018 (12)	−0.0226 (16)	−0.0042 (13)
C12	0.0452 (15)	0.0390 (13)	0.0739 (19)	0.0016 (12)	−0.0192 (14)	0.0019 (13)
C13	0.0386 (12)	0.0341 (11)	0.0466 (13)	0.0009 (10)	0.0053 (10)	0.0031 (10)
C14	0.0381 (13)	0.0320 (12)	0.0724 (18)	−0.0039 (10)	−0.0043 (12)	−0.0003 (11)
C15	0.0381 (13)	0.0376 (13)	0.0692 (18)	0.0013 (10)	−0.0074 (13)	−0.0007 (12)
C16	0.0408 (13)	0.0357 (12)	0.0517 (14)	0.0007 (10)	0.0052 (11)	0.0057 (11)
O11	0.0486 (11)	0.0379 (10)	0.0880 (15)	0.0055 (9)	−0.0130 (11)	0.0024 (10)
O12	0.0483 (11)	0.0317 (9)	0.0721 (13)	0.0001 (8)	−0.0004 (9)	0.0024 (8)
O21	0.0479 (11)	0.0665 (14)	0.0564 (12)	−0.0085 (10)	−0.0011 (9)	0.0004 (10)
C21	0.072 (2)	0.092 (3)	0.066 (2)	0.011 (2)	−0.0128 (18)	0.0078 (19)
C22	0.125 (5)	0.201 (7)	0.059 (2)	0.049 (4)	−0.002 (3)	0.000 (3)

### Geometric parameters (Å, º)

**Table:**

Cd1—N1	2.220 (3)	C12—H12	0.9300
Cd1—O11	2.247 (2)	C13—C14	1.379 (4)
Cd1—N11^i^	2.275 (2)	C13—C16	1.508 (3)
Cd1—O21	2.351 (2)	C14—C15	1.378 (4)
Cd1—O12	2.583 (2)	C14—H14	0.9300
Cd1—S1^ii^	2.6644 (9)	C15—H15	0.9300
Cd1—C16	2.746 (3)	C16—O12	1.245 (3)
N1—C1	1.144 (4)	C16—O11	1.258 (3)
C1—S1	1.635 (3)	O21—C21	1.422 (4)
S1—Cd1^iii^	2.6644 (9)	O21—H1O1	0.8199
N11—C11	1.331 (4)	C21—C22	1.441 (7)
N11—C15	1.331 (3)	C21—H21A	0.9700
N11—Cd1^iv^	2.275 (2)	C21—H21B	0.9700
C11—C12	1.380 (4)	C22—H22A	0.9600
C11—H11	0.9300	C22—H22B	0.9600
C12—C13	1.365 (4)	C22—H22C	0.9600
			
N1—Cd1—O11	108.39 (10)	C12—C13—C14	117.8 (2)
N1—Cd1—N11^i^	106.05 (11)	C12—C13—C16	119.9 (2)
O11—Cd1—N11^i^	145.09 (8)	C14—C13—C16	122.3 (2)
N1—Cd1—O21	84.95 (9)	C15—C14—C13	119.2 (2)
O11—Cd1—O21	88.73 (8)	C15—C14—H14	120.4
N11^i^—Cd1—O21	88.70 (8)	C13—C14—H14	120.4
N1—Cd1—O12	161.97 (10)	N11—C15—C14	123.0 (2)
O11—Cd1—O12	53.60 (7)	N11—C15—H15	118.5
N11^i^—Cd1—O12	91.82 (7)	C14—C15—H15	118.5
O21—Cd1—O12	93.19 (7)	O12—C16—O11	122.9 (2)
N1—Cd1—S1^ii^	89.26 (8)	O12—C16—C13	120.5 (2)
O11—Cd1—S1^ii^	94.89 (7)	O11—C16—C13	116.6 (2)
N11^i^—Cd1—S1^ii^	91.09 (6)	O12—C16—Cd1	69.29 (14)
O21—Cd1—S1^ii^	173.91 (6)	O11—C16—Cd1	53.83 (13)
O12—Cd1—S1^ii^	92.90 (5)	C13—C16—Cd1	169.39 (19)
N1—Cd1—C16	135.24 (11)	C16—O11—Cd1	99.30 (16)
O11—Cd1—C16	26.87 (8)	C16—O12—Cd1	83.93 (15)
N11^i^—Cd1—C16	118.58 (8)	C21—O21—Cd1	130.7 (2)
O21—Cd1—C16	92.37 (8)	C21—O21—H1O1	112.8
O12—Cd1—C16	26.78 (7)	Cd1—O21—H1O1	113.7
S1^ii^—Cd1—C16	93.06 (6)	O21—C21—C22	114.9 (3)
C1—N1—Cd1	168.3 (3)	O21—C21—H21A	108.5
N1—C1—S1	179.2 (3)	C22—C21—H21A	108.5
C1—S1—Cd1^iii^	96.29 (10)	O21—C21—H21B	108.5
C11—N11—C15	117.4 (2)	C22—C21—H21B	108.5
C11—N11—Cd1^iv^	120.52 (17)	H21A—C21—H21B	107.5
C15—N11—Cd1^iv^	121.17 (18)	C21—C22—H22A	109.5
N11—C11—C12	122.7 (3)	C21—C22—H22B	109.5
N11—C11—H11	118.7	H22A—C22—H22B	109.5
C12—C11—H11	118.7	C21—C22—H22C	109.5
C13—C12—C11	119.8 (3)	H22A—C22—H22C	109.5
C13—C12—H12	120.1	H22B—C22—H22C	109.5
C11—C12—H12	120.1		

Symmetry codes: (i) −*x*+1, *y*+1/2, −*z*+3/2; (ii) *x*+1, *y*, *z*; (iii) *x*−1, *y*, *z*; (iv) −*x*+1, *y*−1/2, −*z*+3/2.

### Hydrogen-bond geometry (Å, º)

**Table:**

*D*—H···*A*	*D*—H	H···*A*	*D*···*A*	*D*—H···*A*
O21—H1*O*1···O12^iii^	0.82	1.89	2.703 (3)	172

Symmetry code: (iii) *x*−1, *y*, *z*.
